# Two Novel Space-Time Coding Techniques Designed for UWB MISO Systems Based on Wavelet Transform

**DOI:** 10.1371/journal.pone.0167990

**Published:** 2016-12-13

**Authors:** Amira Ibrahim Zaki, Ehab F. Badran, Said E. El-Khamy

**Affiliations:** Department of Electronics and Communications Engineering, Arab Academy for Science, Technology, and Maritime Transport, Alexandria, Egypt; West Virginia University, UNITED STATES

## Abstract

In this paper two novel space-time coding multi-input single-output (STC MISO) schemes, designed especially for Ultra-Wideband (UWB) systems, are introduced. The proposed schemes are referred to as wavelet space-time coding (WSTC) schemes. The WSTC schemes are based on two types of multiplexing, spatial and wavelet domain multiplexing. In WSTC schemes, four symbols are transmitted on the same UWB transmission pulse with the same bandwidth, symbol duration, and number of transmitting antennas of the conventional STC MISO scheme. The used mother wavelet (MW) is selected to be highly correlated with transmitted pulse shape and such that the multiplexed signal has almost the same spectral characteristics as those of the original UWB pulse. The two WSTC techniques increase the data rate to four times that of the conventional STC. The first WSTC scheme increases the data rate with a simple combination process. The second scheme achieves the increase in the data rate with a less complex receiver and better performance than the first scheme due to the spatial diversity introduced by the structure of its transmitter and receiver. The two schemes use Rake receivers to collect the energy in the dense multipath channel components. The simulation results show that the proposed WSTC schemes have better performance than the conventional scheme in addition to increasing the data rate to four times that of the conventional STC scheme.

## Introduction

The UWB transmission is a developed short range technique which transmits information with a very high data rate. This is due to the fact that the UWB system uses a train of very short duration pulses, in the range of Nano second (i.e. transmitting over a very wide bandwidth), to transmit the data symbols [1]. The characteristics of these ultra-short and the provided high data rate made the UWB transmission the preferred choice in many fields like the military and biomedical fields and in many systems like Wireless Local Area Network (WLAN) and Personal Local Area Network (PAN) [2], [3].

The Federal Communication Committee (FCC) assigned strict regulations to allow the UWB transmission. These regulations permit the UWB systems to transmit over an ultra-wide bandwidth without affecting the services and systems previously allocated in the same spectrum [4]. The FCC regulations states that, the UWB systems are permitted to transmit over the frequency band that ranges between 3.1 and 10.6 GHz, and the UWB pulses are allowed to be transmitted with a very low transmission power -41.3dBm/MHz to overcome the interference with previously allocated systems in the same band [4], [5], [6]. The FCC also defined the UWB signal as the signal that have a -10dB bandwidth ≥ 500MHz or fractional bandwidth > 20%.

The strict FCC regulations on UWB systems and the effect of the fading channel which is extremely frequency selective, limit the achievable data rate and transmission range of the UWB system. The UWB channel has the characteristics of being a dense multipath channel. Where, this channel is dense with resolvable multipath components due to the transmission of ultra-short pulses. These resolvable multipath components can be captured using a Rake receiver to enhance the performance of the UWB system using number of correlators or fingers (i.e. performing multipath diversity) [4], [6]. For all that, the Rake receiver improves the performance of the UWB system, still the performance of the system is poor due to the FCC power limitation. To overcome the power limitation, the UWB transmission is implemented and studied using different systems. UWB MISO systems are presented to obtain multi data stream (MS) transmission in [7], [8]. It is also studied with a UWB space–time coding (STC) technique based on Alamouti’s scheme [9], [10] using maximum ratio combiner (MRC) Rake receiver in [11] and [12]. This STC technique makes use of multipath diversity in addition to spatial diversity, and thus increases system capacity or enhances the BER performance [13], [14]. In [15], by utilizing interference alignment and channel diagonalization, the authors designed an interference mitigation scheme which was implemented through a local two-step precoder-and-receiver design process. This scheme is to suppress inter-user and intra-user interferences and it can be can be utilised in several wireless systems, such as MIMO, LTE-A, and fifth generation systems.

Many researches on UWB system have included the use of the wavelet transform (WT) and wavelet packet transform (WPT), after it has been used widely in other wireless communication systems [16]. The WT was studied in different parts and techniques of the wireless communication systems. In [17], the WT is used to present a new modulation technique Wavelet Shift Keying (WSK). The proposed WSK modulation technique is observed as an extension of the “Wavelet based Orthogonal Frequency Division Multiplexing (OFDM)” studied in [18] to enhance the performance of the conventional OFDM. The Discrete Wavelet Transform (DWT) and Discrete Multi-Wavelet Transform (DMWT) are also studied with the conventional OFDM systems to reduce the interference level and increase spectral efficiency as shown in [19]. The DMWT–OFDM proposed in [19] presents, a decrease in the bit error rate (BER) and an increase in the signal to noise ratio (SNR) when compared to the conventional OFDM. In [20], DWT was applied as a processing tool to accomplish joint antenna selection over correlated MIMO channels.

Recently, the WT was used in the UWB communication systems to enhance the performance of the system and fulfil the restrictions of the FCC. The WT is used in [21] and [22] to define new UWB pulse shapes to satisfy the FCC limits on the power and bandwidth, in addition to enhancing the spectral efficiency. The WT is also introduced in [23] as a technique to reject the narrowband and wideband interferences.

On the other hand, the WT is introduced in [24] as a mean of analyzing the UWB signal and extracting it from the background noise. The proposed technique in [24] succeeded to detect the desired signal and enhance the BER performance. In UWB communication systems a new Rake receiver based on continuous WT is proposed in [25] where the Rake correlators uses scaled wavelet versions as the template pulses instead of the time delayed template pulses used in conventional Rake. The proposed Rake in [25] was examined and compared to the conventional Rake. The simulations and results in [25] show that the continuous WT Rake receiver outperforms the conventional Rake in the BER performance with less number of correlators.

In this paper two WSTC schemes (WSTC-I) and (WSTC-II) are proposed to increases the data rate to four times that of a conventional STC introduced in [11], [12], in addition to enhancing the system performance without increasing the bandwidth, pulse duration or number of transmitting antenna.

The first scheme WSTC-I is based on multiplexing multiple symbols in the wavelet domain of the UWB pulses in addition to the spatial multiplexing offered by using multiple transmitting antennas. The second scheme WSTC-II uses the same benefits of the WSTC-I in addition to introducing spatial diversity gain. The WSTC-II uses the same transmitter of the WSTC-I but with a different code word and different receiver with less complexity. The used mother wavelet (MW) in both schemes is selected to be highly correlated with transmitted pulse shape and such that the multiplexed signal has almost the same spectral characteristics as those of the original UWB pulse. The WSTC schemes use Rake receivers to collect the energy in the dense multipath channel components. The simulation results show that the proposed WSTC schemes have better performance than the conventional scheme.

The paper is organized as follows: In Section 2, the channel model used in the simulation is introduced. Section 3 discusses the system model for a single transmitting and receiving antenna (SISO), and the conventional STC UWB systems in [11], [12]. Section 4 presents the proposed WSTC scheme. Section 5 introduces the simulations and results. Finally, the conclusions are shown in Section 6.

## Channel Model

The channel models used is the modified Saleh-Valenzuela (SV) model [26]. The impulse response of the SV channel model can be expressed in terms of the cluster and multipath rays gains and arrival times as follows [26]

$$h(t)=X\sum_{k=0}^{K}\sum_{m=0}^{M}\alpha (k,m)\delta (t-T(k)-\tau (k,m))$$

Where

- *K* is the number of clusters in the channel and *M* is the number of multipath rays per cluster.
- *δ*(*t*) is the Dirac function.
- *α*(*k*,*m*) is the multipath gain coefficient of the *m*^*th*^ ray in the *k*^*th*^ cluster.
- *T*(*k*) is the delay of the *k*^*th*^ cluster.
- *τ*(*k*,*m*) is the delay of the the *m*^*th*^ ray in the *k*^*th*^ cluster relative to the *k*^*th*^ cluster arrival time *T*(*k*).
- *X* represents the log-normal shadowing.

The gain of the channel due to measurements as states in [26] best fit the log-normal distribution with a cluster and ray decaying factors *Γ* and *γ* respectively, while the arrival times of the clusters and the rays included fit Poisson distribution with arrival rates *Λ* and *λ* respectively. The parameters of the different line of sight (LOS) and non-line of sight (NLOS) channels are shown in Table 1 [26].

**Table 1:** Channels model parameters as presented in the IEEE802.15.3a report.

Model Parameters	Multipath Channels			
	*CM1*	*CM2*	*CM3*	*CM4*
Channel Condition	LOS	NLOS	NLOS	Extreme NLOS
Range	0–4m	0–4m	1–10m	0–10m
Cluster arrival rate (Λ [1/nsec])	0.0233	0.4	0.0667	0.0667
Ray arrival rate (*λ* [1/nsec])	2.5	0.5	2.1	2.1
Cluster decay factor (Γ)	7.1	5.5	14	24
Ray decay factor (*γ*)	4.3	6.7		12

For simplicity of the analysis and without loss of generality, a single cluster is assumed, thus the impulse response of the channel can be simplified as:

$$h(t)=\sum_{m=0}^{M-1}\alpha (m)\delta (t-\tau (m)),$$ (1)

where, *M* is the number of multipath components, while, *α*(*m*) and *τ*(*m*) are the gain and the delay of the *m*^*th*^ path respectively.

## System Model

This section, presents the system model used in this paper for a UWB, SISO and conventional STC MISO systems, for peer-to-peer communication. In the UWB communications binary symbols *s* = ±1 are transmitted over a train of Nano second duration pulses. The system has *N*_*t*_ transmit and *N*_*r*_ receive antennas. The binary symbol is pulse shaped by motorcycle pulse *w*(*t*). Then, the symbols are modulated by pulse amplitude modulation (PAM) and transmitted repeatedly over *N*_*f*_ frames each of time duration *T*_*f*_ (*T*_*s*_ = *N*_*f*_*T*_*f*_, where *T*_*s*_ is the symbol duration). The duration of the monocycle pulse *w*(*t*) is given by *T*_*w*_ and has a typical duration between 0.2–2ns, resulting in a transmission over an ultra-wide bandwidth. Modelling the system in this paper is based on the assumption that the channel impulse response (CIR) is known at the receiver and the channel is constant for a block of symbols (quasi-static channel).

### SISO Scheme

If a single transmit and receive antennas are assumed (SISO), and PAM modulation, the transmitted waveform for the binary symbol *s* is given by

$$S(t)=s\sqrt{\frac{E}{N_{f}}}\sum_{n_{f}=0}^{N_{f}-1}w(t-n_{f}T_{f}),$$ (2)

where, *E* is the symbol energy, and pulse shape *w*(*t*) is of unit energy. The multipath channel can be expressed in terms of multipath gains and delays as stated in (1), where *τ*(*m*) > *τ*(*m*−1), and *T*_*m*_ = *τ*(*M*−1) is the maximum delay spread of the dense multipath channel. To avoid the inter symbol interference (ISI), simply choose *T*_*f*_ ≥ *T*_*m*_ + *T*_*w*_. The modelled multipath fading channel is assumed to be quasi-static (i.e. constant during a block of symbols).

A Rake receiver is used at the receiver to collect multipath diversity. It has *L* fingers (matched filters), where *L* ≤ *M*, and uses *w*(*t*) as the correlator reference template with an autocorrelation function *R*_*w*_(*τ*)[11].

### Conventional STC MISO Scheme

The conventional STC-I MISO system presented in [11] and [12] (which will be referred to as conventional STC) is based on transmitted two space-time coded symbols over the even and odd frames of a single symbol duration. The encoded symbols are transmitted from two transmitting antennas and received by single antenna, then a Rake receiver and an MRC are used to combine the encoded data sent over the even and odd frames and estimate the transmitted symbols (for more details refer to [11]). For an STC MISO system the output of the first transmit antenna during each symbol *T*_*s*_ = *N*_*f*_*T*_*f*_ is given by [12]

$$S_{0}(t)=s\sqrt{\frac{E}{2N_{f}}}\sum_{n_{f}=0}^{N_{f}-1}{\left(-1\right)}^{n_{f}}w(t-n_{f}T_{f}),$$ (3)

and the output of the second transmit antenna is:

$$S_{1}(t)=s\sqrt{\frac{E}{2N_{f}}}\sum_{n_{f}=0}^{N_{f}-1}w(t-n_{f}T_{f})$$ (4)

## The Proposed Wavelet Space-Time Coding Technique

This section illustrates the transmitters, the code words, the receivers and analytical systems models of the proposed wavelet space-time coding techniques (WSTC). The WSTC combines four symbols using the inverse discrete wavelet transform (IDWT) and multiple transmitting antennas (multiplexing in frequency and space) and send them on the same bandwidth and same symbol duration of the conventional STC. This section presents two WSTC schemes as follow.

### Wavelet Space-Time Coding Scheme-I (WSTC-I)

**Fig 1:**
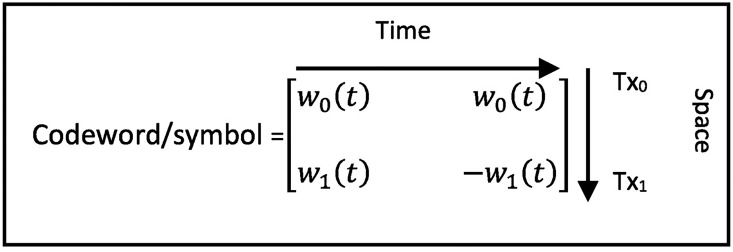
WSTC-I code word per symbol for *N*_*f*_ = 2.

**Fig 2:**
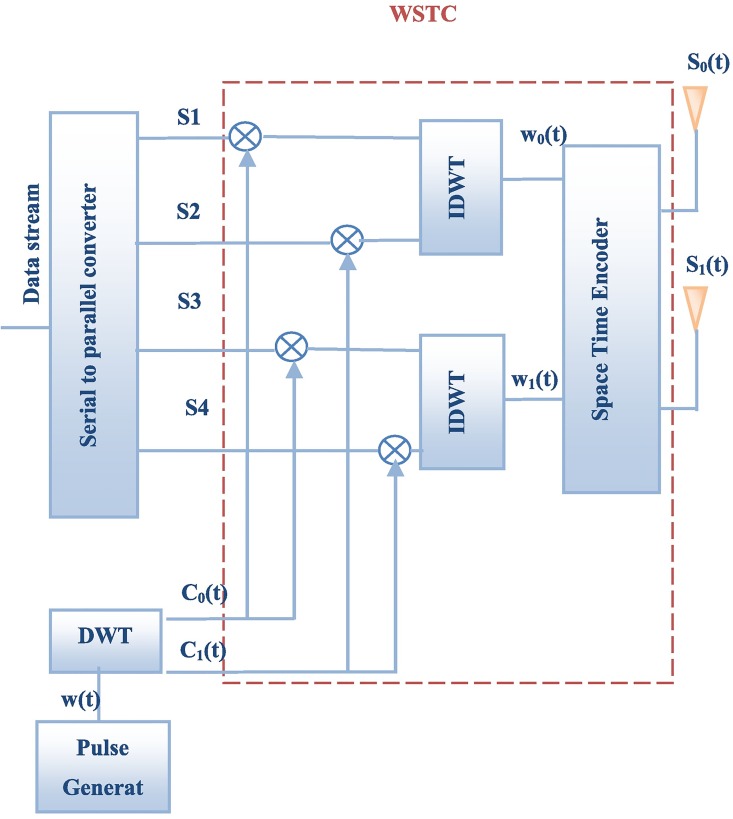
WSTC transmitter.

**Fig 3:**
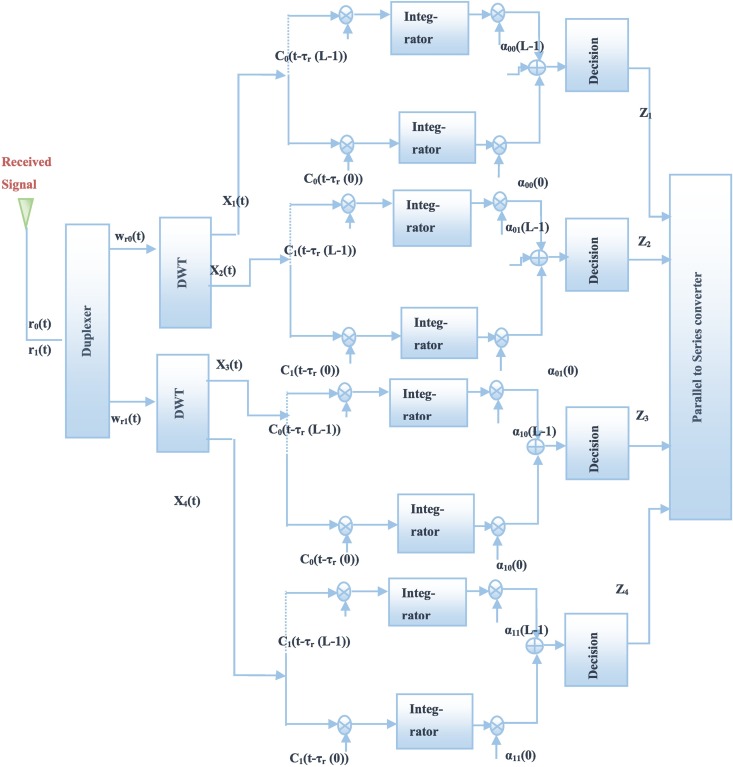
WSTC-I receiver.

The WSTC-I introduced in this section increases the transmission rate without increasing the bandwidth or the transmission symbol duration. It sends half of the symbols on the same transmitted pulse on different transmitting antennas. The WSTC-I code word is shown in Fig 1 while the transmitter and receiver are shown in Fig 2 and Fig 3 respectively. For a MISO WSTC-I (*N*_*t*_ = 2 and *N*_*r*_ = 1), the outputs of the first transmit antenna and the second transmit antenna during each symbol *T*_*s*_ = *N*_*f*_*T*_*f*_ are given by

$$S_{j}(t)=\sqrt{\frac{2E}{N_{f}}}\sum_{n_{f}=0}^{N_{f}-1}{\left(-1\right)}^{\left(j.n_{f}\right)}w_{j}(t-n_{f}T_{f}),\;j=0,1$$ (5)

where *w*_0_(*t*) and *w*_1_(*t*) are the transmitted pulse *w*(*t*) embedded with *s*_1_, *s*_2_ and *s*_3_, *s*_4_ respectively. The waveform *w*_0_(*t*) is the IDWT of the approximation waveform *x*_1_(*t*) and the detail waveform *x*_2_(*t*), while *w*_1_(*t*) is the IDWT of *x*_3_(*t*) and *x*_4_(*t*) respectively. ${\left\{x_{i}(t)\right\}}_{i=1}^{4}$ can be expressed as

$$x_{i}(t)=s_{i}\sqrt{\frac{E}{N_{f}}}C_{(i+1)\;\mathrm{mod}\;2}(t),\;i=1,2,3,4.$$

The two waveforms *C*_0_(*t*) and *C*_1_(*t*) are the approximation and detail components of the transmitted pulse *w*(*t*). Note that, in this scheme, the first and second symbols are transmitted from the first antenna, while the third and the fourth symbols are transmitted from the second one, during the same symbol duration *T*_*s*_. The received noisy signals per even frame *r*_0_(*t*) and odd frame *r*_1_(*t*) for *M* multipath components are presented as follows

$$\begin{array}{l} r_{l}(t)=S_{0}(t)*h_{0}(t)+S_{1}(t)*h_{1}(t)+\eta_{l}(t),\;l=0,1\\ \;=\sqrt{\frac{2E}{N_{f}}}(w_{0}(t)*h_{0}(t)+{\left(-1\right)}^{l}w_{1}(t)*h_{1}(t))+\eta_{l}(t)\\ \;=\sqrt{\frac{2E}{N_{f}}}\sum_{m=0}^{M-1}\left(\alpha_{0}(m)w_{0}(t-\tau_{0}(m))+{\left(-1\right)}^{l}\alpha_{1}(m)w_{1}(t-\tau_{1}(m)\right))+\eta_{l}(t), \end{array}$$ (6)

where *h*_*i*_(*t*) is the impulse response between the *j*^*th*^ transmitting and the receiving antenna, *α*_*j*_(*m*) and *τ*_*j*_(*m*) are the gain and the delay of the *m*^*th*^ multipath component. The two symbols *η*_0_(*t*) and *η*_1_(*t*) are the additive white Gaussian noise (AWGN) with zero mean and *σ*^2^ variance of the even and odd frames respectively.

The even and odd frames are combined using a combiner to generate waveforms *w*_*r*0_(*t*) and *w*_*r*1_(*t*), which are the estimated waveforms of the transmitted ones *w*_0_(*t*) and *w*_1_(*t*) respectively. The estimated waveforms are

$$\begin{array}{l} w_{r0}(t)=r_{0}(t)+r_{1}(t)\\ \;=2\sqrt{\frac{2E}{N_{f}}}\sum_{m=0}^{M-1}\left(\alpha_{0}(m)w_{0}(t-\tau_{0}(m\right)))+\eta_{0}(t)+\eta_{1}(t) \end{array}$$ (7)

and

$$\begin{array}{l} w_{r1}(t)=r_{0}(t)-r_{1}(t)\\ \;=2\sqrt{\frac{2E}{N_{f}}}\sum_{m=0}^{M-1}\left(\alpha_{1}(m)w_{1}(t-\tau_{1}(m\right)))+\eta_{1}(t)-\eta_{o}(t), \end{array}$$ (8)

where, the two noise components, *η*_0_(*t*)−*η*_1_(*t*) and *η*_0_(*t*)+*η*_1_(*t*), both have variance equal to 2*σ*^2^. By applying the DWT to the estimated waveforms, their approximation and detail components (carrying the symbols) are *X*_1_(*t*) and *X*_2_(*t*) respectively for *w*_*r*0_(*t*) and, *X*_3_(*t*) and *X*_4_(*t*) for, *w*_*r*1_(*t*) are obtained as

$$X_{i}(t)=2s_{i}\sqrt{\frac{E}{N_{f}}}\sum_{m=0}^{M-1}\left(\alpha_{\left\lfloor \frac{i}{3}\right\rfloor }(m)C_{(i+1)\mathrm{mod}2}(t-\tau_{\left\lfloor \frac{i}{3}\right\rfloor }(m\right))+\varepsilon_{i}(t),$$ (9)

where *ε*_1_(*t*) and *ε*_3_(*t*) are the approximation components of *η*_0_(*t*)+*η*_1_(*t*) and *η*_0_(*t*)−*η*_1_(*t*) successively while *ε*_2_(*t*) and *ε*_4_(*t*) are their detail components. The approximation and detail components have variance equal to 2*σ*^2^ (as the detail and approximate of the DWT represents the high pass and low pass components of the signal after the down-sampling by 2, thus the power spectral density will not be changed after the DWT and so the variance). The approximation and detail components pass through a Rake receiver. The output per Rake finger for each component is

$$\begin{array}{l} x_{i}(l)=(\int_{0}^{T_{f}}\left(2s_{i}\sqrt{\frac{E}{N_{f}}}\sum_{m=0}^{M-1}\alpha_{\left\lfloor \frac{i}{3}\right\rfloor }(m)C_{(i+1)\mathrm{mod}2}(t-\tau_{\left\lfloor \frac{i}{3}\right\rfloor }(m))C_{(i+1)\mathrm{mod}2}(t-\tau_{r}(l)\right)\\ \;+\varepsilon_{1}(t)C_{(i+1)\mathrm{mod}2}(t-\tau_{r}(l)))dt)\alpha_{r\left\lfloor \frac{i}{3}\right\rfloor ,(i+1)\mathrm{mod}2}(l)\\ \;=(2s_{i}\sqrt{\frac{E}{N_{f}}}\sum_{m=0}^{M-1}\left(\alpha_{\left\lfloor \frac{i}{3}\right\rfloor }(m)Rw_{(i+1)\mathrm{mod}2}(\tau_{r}(l)-\tau_{\left\lfloor \frac{i}{3}\right\rfloor }(m\right)))\\ \;+\int_{0}^{T_{f}}\varepsilon_{i}(t)C_{(i+1)\mathrm{mod}2}(t-\tau_{r}(l))dt).\alpha_{r\left\lfloor \frac{i}{3}\right\rfloor ,(i+1)\mathrm{mod}2}(l)\\ \;=2s_{i}\sqrt{\frac{E}{N_{f}}}\alpha^{2}{}_{r\left\lfloor \frac{i}{3}\right\rfloor ,(i+1)\mathrm{mod}2}(l)+(\int_{0}^{T_{f}}\varepsilon_{i}(t)C_{(i+1)\mathrm{mod}2}(t-\tau_{r}(l))dt)\alpha_{r\left\lfloor \frac{i}{3}\right\rfloor ,(i+1)\mathrm{mod}2}(l), \end{array}$$ (10)

where *τ*_*r*_(*l*) is the delay of the *l*^*th*^ Rake finger, *Rw*_0_(*t*) is the autocorrelation of *C*_0_(*t*) and *Rw*_1_(*t*) is the autocorrelation of *C*_1_(*t*). The reference pulses of the Rake receiver are assumed to be of unit energy, and

$$\alpha_{rj,d}(l)=\sum_{m=0}^{M-1}\left(\alpha_{j}(m)Rw_{d}(\tau_{r}(l)-\tau_{j}(m\right)),\;j=0,1\;\text{and}\;d=0,1$$ (11)

By summing up the Rake finger outputs and the *N*_*f*_ frames, the resulting decision statistics equivalent to symbol *s*_1_, *s*_2_ and *s*_3_, *s*_4_ are given by

$$\begin{array}{l} Z_{i}=s_{i}\sqrt{N_{f}E}\sum_{l=0}^{L-1}\alpha^{2}{}_{r\left\lfloor \frac{i}{3}\right\rfloor ,(i+1)\mathrm{mod}\;2}(l)+\sum_{nf=0}^{\frac{Nf}{2}-1}\sum_{l=0}^{L-1}(\int_{0}^{T_{f}}\varepsilon_{i}(t)C_{(i+1)\mathrm{mod}\;2}(t-\tau_{r}(l))dt)\alpha_{r\left\lfloor \frac{i}{3}\right\rfloor ,(i+1)\mathrm{mod}\;2}(l)\\ \;=s_{i}\sqrt{N_{f}E}{\text{E}}_{\left\lfloor \frac{i}{3}\right\rfloor ,(i+1)\mathrm{mod}\;2}+\rho_{i}, \end{array}$$ (12)

where *E*_*i*,*d*_ are the energy captured by the Rake receiver *ρ*_*i*_ is the noise component which has a variance *N*_*f*_*σ*^2^*E*_*i*,(*i*+1)mod 2_. When the maximum-likelihood (ML) detector is used, the conditional the BER is given by

$$P(e/{\left\{\alpha_{rj,d}(l)\right\}}_{l=0}^{L-1})=Q(\sqrt{SNR_{t}\;E_{i,d}})),$$ (13)

where *SNR*_*t*_ is the transmitted signal to noise ratio, and Q is the Gaussian error function. Note that the Rake receiver is composed of a number of correlator fingers, where the reference waveforms are delayed versions of the approximation and detail waveforms of *w*(*t*). The delays of the Rake fingers are fixed and equivalent to the delays of the selected multipath components.

The mother wavelet (MW) transform function used is chosen to be with high similarity with the transmitted pulse and poor with the noise. Thus, at the correlators output, high correlation is obtained with the transmitted pulse and low with the background noise. Also while choosing MW it must be taken into consideration that the pulse spectrum characteristics do not vary much when it is embedded with the two transmitted symbols per antenna (multiplexed and carried on the approximation and detail waveforms of *w*(*t*)).

### Wavelet Space-Time Coding Scheme-II (WSTC-II)

**Fig 4:**
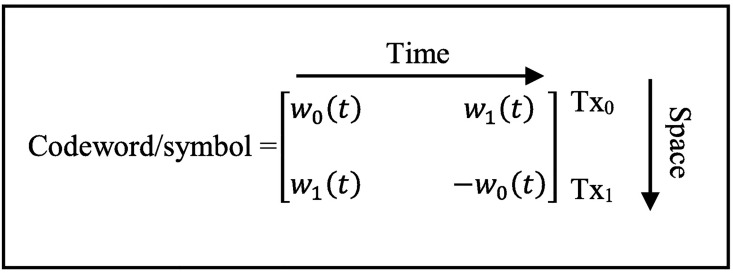
WSTC-II code word per symbol for *N*_*f*_ = 2.

**Fig 5:**
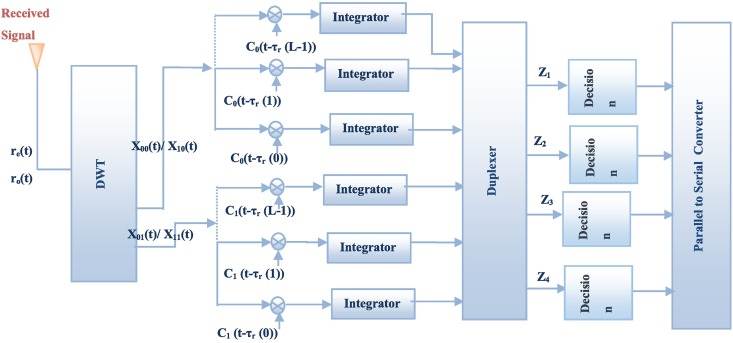
WSTC-II receiver.

The WSTC-II introduced in this section makes use of the same benefits of the WSTC-I in addition to introducing a spatial diversity gain. Thus, this scheme introduces frequency spatial multiplexing and diversity gain. Another benefit of WSTC-II over WSTC-I is the reduction of the receiver complexity to the half. The WSTC-II uses the same transmission configuration illustrated in Fig 2 but with the code word in Fig 4. The WSTC-II receiver is demonstrated in Fig 5.

The output of the first transmit antenna during an even frame is given by

$$S_{0}(t)=\sqrt{\frac{2E}{N_{f}}}\sum_{n_{f}=0}^{\frac{N_{f}}{2}-1}w_{0}(t-2n_{f}T_{f}),$$ (14)

and for the second antenna is given by

$$S_{1}(t)=\sqrt{\frac{2E}{N_{f}}}\sum_{n_{f}=0}^{\frac{N_{f}}{2}-1}w_{1}(t-2n_{f}T_{f}).$$ (15)

For the odd frames the output of the first transmitting antenna is

$$S_{0}(t)=\sqrt{\frac{2E}{N_{f}}}\sum_{n_{f}=1}^{\frac{N_{f}}{2}}w_{1}(t-(2n_{f}-1)T_{f}),$$ (16)

while for the second transmitting antenna

$$S_{1}(t)=\sqrt{\frac{2E}{N_{f}}}\sum_{n_{f}=1}^{\frac{N_{f}}{2}}-w_{0}(t-(2n_{f}-1)T_{f}).$$ (17)

Thus, the received signal per even frame *r*_0_(*t*) and the received signal per odd frame *r*_1_(*t*) will be

$$\begin{array}{l} r_{l}(t)=\sqrt{\frac{2E}{N_{f}}}(w_{l}(t)*h_{0}(t)+w_{\left(1-l\right)}(t)*h_{1}(t))+\eta_{l}(t)\;l=0,1\\ =\sqrt{\frac{2E}{N_{f}}}\sum_{m=0}^{M-1}\left(\alpha_{0}(m)w_{l}(t-\tau_{0}(m))+\alpha_{1}(m)w_{\left(1-l\right)}(t-\tau_{1}(m)\right))+\eta_{l}(t), \end{array}$$ (18)

where *η*_0_(*t*) and *η*_1_(*t*) are the additive white Gaussian noise (AWGN) with zero mean and variance of the even and odd frames respectively. By applying the DWT at the receiver, both *r*_0_(*t*) and *r*_1_(*t*) will be split to their approximation and detail waveform components. The approximation component *X*_00_(*t*) and detail *X*_01_(*t*) for *r*_0_(*t*) are given by

$$X_{0d}(t)=\sqrt{\frac{E}{N_{f}}}\sum_{m=0}^{M-1}\left(s_{d+1}\alpha_{0}(m)C_{d}(t-\tau_{0}(m)\right)+s_{d+3}\alpha_{1}(m)C_{d}(t-\tau_{1}(m)))+\eta_{0d}(t)\;d=0,1$$ (19)

and for *r*_1_(*t*)

$$X_{1d}(t)=\sqrt{\frac{E}{N_{f}}}\sum_{m=0}^{M-1}\left(s_{d+3}\alpha_{0}(m)C_{d}(t-\tau_{0}(m\right))-s_{d+1}\alpha_{1}(m)C_{d}(t-\tau_{10}(m)))+\eta_{1d}(t)$$ (20)

where *η*_0*d*_(*t*) and *η*_1*d*_(*t*) are the DWT noise components with *σ*^2^ variance. The output per Rake finger for *X*_00_(*t*), *X*_01_(*t*), *X*_10_(*t*) and *X*_11_(*t*) are respectively given by

$$\begin{array}{l} y_{0d}(l)=\sqrt{\frac{E}{N_{f}}}\sum_{m=0}^{M-1}\left(s_{d+1}\alpha_{0}(m)Rw_{d}(\tau_{r}(l)-\tau_{0}(m\right))\\ \;+s_{d+3}\alpha_{1}(m)Rw_{0}(\tau_{r}(l)-\tau_{1}(m)))+\int_{0}^{T_{f}}\eta_{0d}(t)C_{d}(t-\tau_{r}(l))dt\\ \;=\sqrt{\frac{E}{N_{f}}}(s_{d+1}\alpha_{r0d}(l)+s_{3}\alpha_{r1d}(l))+\xi_{0d}(l) \end{array}$$ (21)

$$\begin{array}{l} y_{1d}(l)=\sqrt{\frac{E}{N_{f}}}\sum_{m=0}^{M-1}\left(s_{d+3}\alpha_{0}(m)Rw_{d}(\tau_{r}(l)-\tau_{0}(m\right))\\ \;-s_{d+1}\alpha_{1}(m)Rw_{A}(\tau_{r}(l)-\tau_{1}(m)))+\int_{0}^{T_{f}}\eta_{1d}(t)C_{d}(t-\tau_{r}(l))dt\\ \;=\sqrt{\frac{E}{N_{f}}}(s_{d+3}\alpha_{r0d}(l)-s_{d+1}\alpha_{r1d}(l))+\xi_{1d}(l), \end{array}$$ (22)

where *ξ*_00/01/10/11_(*l*) are the Rake finger output noise components of the approximation/detail components corresponding to the even and odd frames which have *σ*^2^ variances.The combiner is used on two levels, one level is to combine and sum the Rake fingers, and the second level is to sum the *N*_*f*_ frames. Thus, the resulting decision statistics equivalent to symbol *s*_1_, *s*_2_, *s*_3_ and *s*_4_ are consecutively given by

$$\begin{array}{l} Z_{i}=\sum_{nf=0}^{\frac{Nf}{2}-1}\sum_{l=0}^{L-1}(y_{0\;(\text{i}+1)\text{mod}2}(l).\alpha_{r\left\lfloor \frac{i}{3}\right\rfloor ,(i+1)\mathrm{mod}\;2}(l)+{\left(-1\right)}^{\left(1-\left\lfloor \frac{i}{3}\right\rfloor \right)}y_{1\;(\text{i}+1)\text{mod}2}(l)\alpha_{r(1-\left\lfloor \frac{i}{3}\right\rfloor ),(i+1)\mathrm{mod}\;2}(l))\\ \;=s_{i}\sqrt{\frac{N_{f}E}{4}}({\text{E}}_{0\;(\text{i}+1)\text{mod}2}+{\text{E}}_{1\;(\text{i}+1)\text{mod}2})+\sum_{nf=0}^{\frac{Nf}{2}-1}\sum_{l=0}^{L-1}(\xi_{0\;(\text{i}+1)\text{mod}2}(l).\alpha_{r\left\lfloor \frac{i}{3}\right\rfloor ,\;(\text{i}+1)\text{mod}2}(l)\\ \;+{\left(\text{-}1\right)}^{\left(1-\left\lfloor \frac{i}{3}\right\rfloor \right)}\xi_{1\;(\text{i}+1)\text{mod}2}(l)\alpha_{r(1-\left\lfloor \frac{i}{3}\right\rfloor ),(\text{i}+1)\text{mod}2}(l))\\ \;=s_{i}\sqrt{\frac{N_{f}E}{4}}({\text{E}}_{0\;(\text{i}+1)\text{mod}2}+{\text{E}}_{1\;(\text{i}+1)\text{mod}2})+\upsilon_{i}\;\text{i}=1,2,3,4 \end{array}$$ (23)

where $E_{jd}=\sum_{l=0}^{L-1}\alpha^{2}{}_{rjd}(l)$ (*j* = 0,1, *d* = 0,1) and *v*_*i*_ are the noise components in the decision statistics which have variances equal to *N*_*f*_*σ*^2^(*E*_0*d*_ + *E*_1*d*_)/2. When the maximum-likelihood (ML) detector is used, the conditional the BER is given by

$$P(e/{\left\{\alpha_{rpa/d}(l)\right\}}_{l=0}^{L-1})=Q(\sqrt{\frac{SNR_{t}}{2}(E_{0d}+E_{1d}})\;d=0,1$$ (24)

By comparing the conditional BER of WSTC-I and WSTC-II schemes, it is concluded that WSTC-II scheme introduces a diversity gain (a symbol is received from two channels not only one), this is in addition to the reduction in receiver complexity. Although WSTC-II scheme outperforms WSTC-I scheme but still WSTC-I scheme combination process is simpler than that of WSTC-II.

## Simulation and Results

In this section, the simulation results and comparison between different MISO UWB systems applying the conventional STC, the WSTC-I and the WSTC-II are illustrated. In order to have a fair comparison of the performance of systems with different bit rates, a figure of merit *F* [1/(bit/sec/Hz)] is used for comparing their performances, which is given by

$$F=\frac{BER}{Bandwidth\;\text{Efficiency}}=\frac{BER.Bandwidth}{\text{Bit}\;\text{Rate}}$$

**Fig 6:**
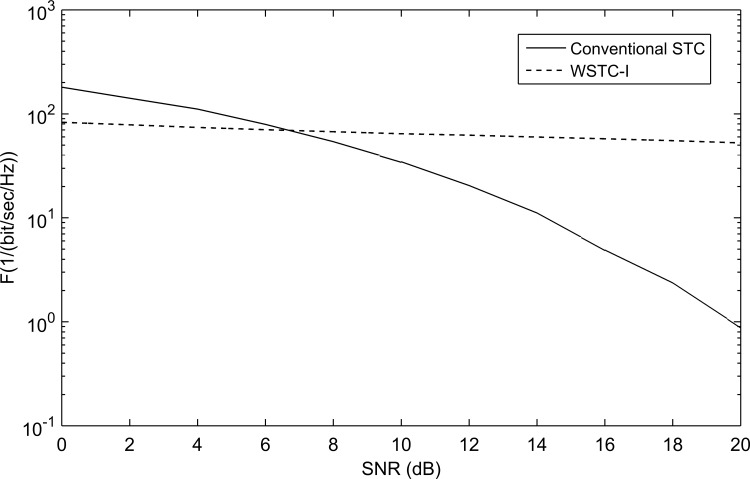
The comparison between the performance of conventional STC and WSTC-I using 2^nd^ derivative Gaussian pulse as a transmission pulse and Coiflet 3 as a MW for *L* = 4 Rake fingers.

As stated before the channel used is the modified SV-model. The used symbol duration *T*_*s*_ = 200nse, with *N*_*f*_ = 2, i.e. *T*_*f*_ == 100 nsec. The transmission monocycle pulse *w*(*t*) used is of pulse width 0.5nsec and unit energy. This configuration eliminates the ISI as *T*_*f*_ ≥ *T*_*m*_ + *T*_*w*_ (where *T*_*m*_ = 99 nsec). The transmission monocycle pulse *w*(*t*) used was first chosen to be the second derivative of the Gaussian function. It showed a very bad performance when embedded (or multiplexed in the IWT domain) with the symbols using different mother wavelets. Fig 6 shows the performance of WSTC-I verses conventional STC using the second derivative Gaussian pulse as a transmission pulse, and Coiflet 3 as the MW for embedding the symbols for *L* = 4. The transmitted pulse is re-shaped to enhance the performance and to keep the spectrum limitations of the FCC. The *w*(*t*) is then taken to be the wavelet detail component of the 2^nd^ derivative Gaussian pulse using MW’s with high similarity with the 2^nd^ derivative Gaussian pulse. The examined MW’s are wavelets with high resembles with the transmitted pulse *w*(*t*) like the Symlets and Coiflets families [16], [27], [28], and [29].

### WSTC-I Results

**Fig 7:**
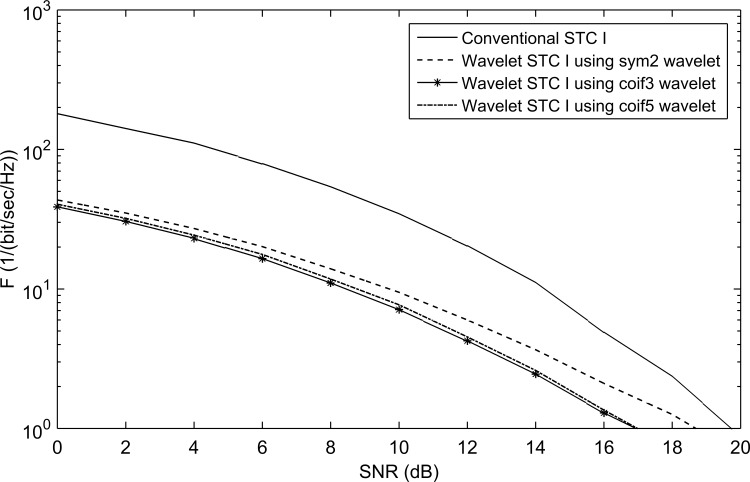
Comparison in performance of the conventional STC and WSTC-I using the detail component of the 2^nd^ derivative Gaussian pulse as the transmission pulse with different MW’s and *L* = 4 Rake fingers.

**Fig 8:**
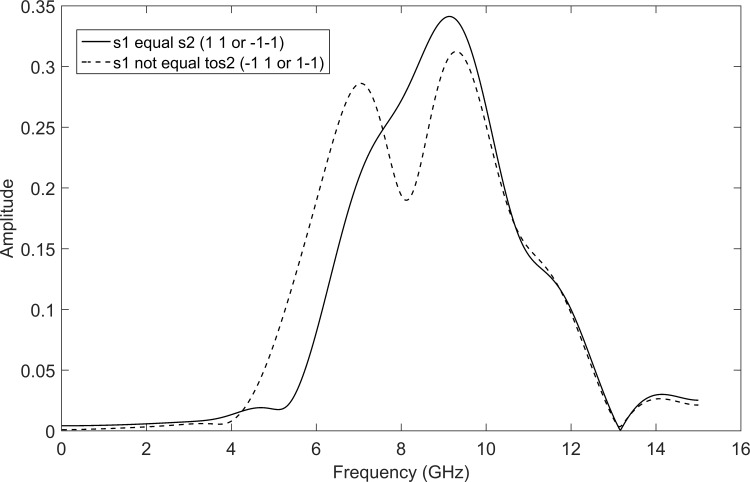
The spectrum of the transmitted pulse *w*_0_(*t*) after the symbols are multiplexed in the wavelet domain (same for *w*_0_(*t*)) using MW Coiflet 5.

**Fig 9:**
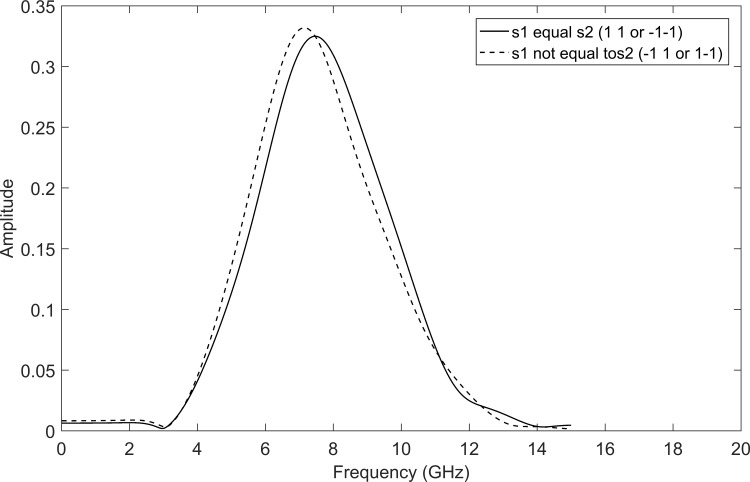
The spectrum of the transmitted pulse *w*_0_(*t*) after the symbols are multiplexed in the wavelet domain (same for *w*_1_(*t*)) using MW Coiflet 3.

Fig 7 shows a comparison of *F* performance of the WSTC-I using different MW’s. It is concluded from Fig 7 that Coiflet 5 presents nearly the same performance as Coiflet 3, but if the MW Coiflet 5 is used, the transmitted pulse will not be able to keep the same bandwidth and spectrum shape after multiplexing the symbols in the wavelet domain (i.e. after the IDWT process in the transmitter) as shown in Fig 8. Thus, the MW Coiflet 3 is preferred due to its good performance, keeping the spectrum characteristics approximately the same when embedded with different symbols and restrict to the FCC spectrum regulations. This can be concluded from Fig 9, which illustrates the spectrum of the transmitted pulse (*w*_0_(*t*) or *w*_1_(*t*)) after being embedded with different symbols (*s*_1_, *s*_2_, *s*_3_ and *s*_4_). Notice that the spectrum of the pulse *w*(*t*) (i.e. before embedding the symbols) is the same as the case *s*_1_ = *s*_2_ in Fig 9.

**Fig 10:**
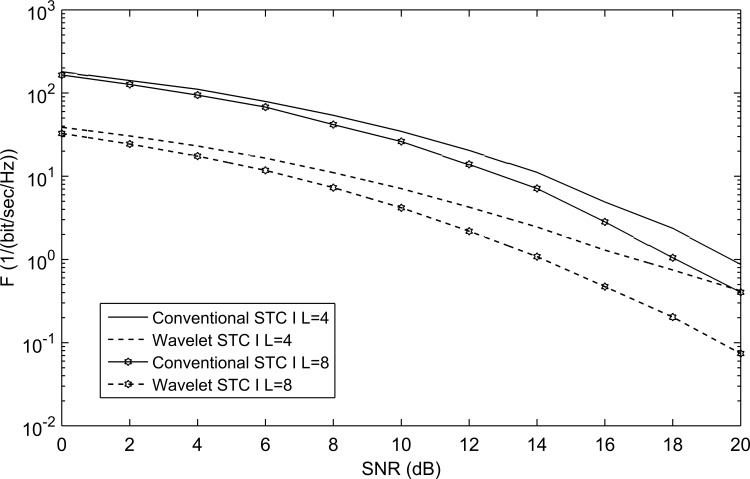
Comparing the performance of conventional STC and WSTC-I UWB system using Coiflet 3 MW with *L* = 4 and *L* = 8 Rake receiver.

The performance of the proposed WSTC-I using Coiflet 3 MW is compared to the conventional STC using *L* = 4 in Fig 10. Fig 10 shows that in addition to the increase in transmission rate the WSTC-I outperforms the conventional STC for *L* = 4 by about 3dB for low SNR (required by the FCC regulations). This is due to the high correlation of the reference pulse with the signal and low correlation with the noise, in addition to performing WT at the receiver which smooth the signal and reduce the noise level. The performance is also illustrated for *L* = 8 (increasing the multipath gain) in the same and the WSTC-I also outperforms the conventional STC by 4dB. It is also concluded from Fig 10 that the proposed WCTC-I scheme with 4 Rake fingers gives better performance than the conventional STC with 8 Rake fingers, i.e. WSTC-I scheme presents better performance with low complex Rake receiver.

**Fig 11:**
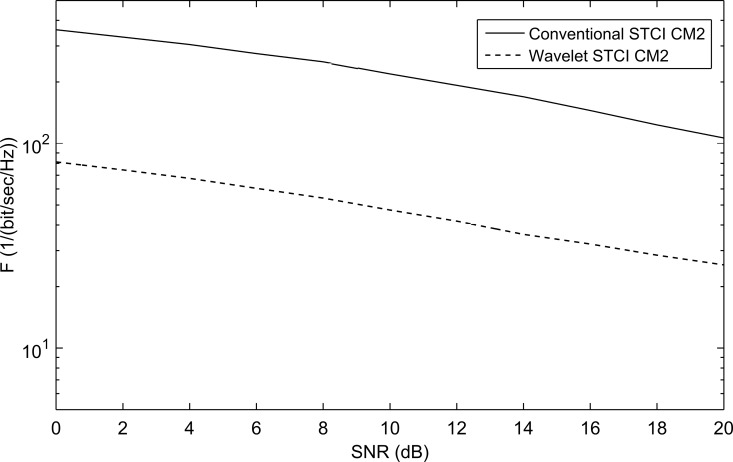
The performance of conventional STC and WSTC-I UWB system using Coiflet 3 MW and *L* = 4 Rake receiver for the CM2 model.

**Fig 12:**
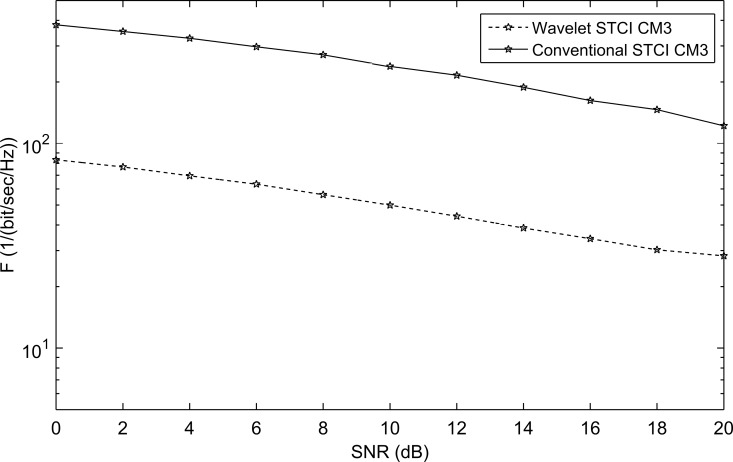
The performance of conventional STC and WSTC-I UWB system using Coiflet 3 MW and *L* = 4 Rake receiver for the CM3 model.

**Fig 13:**
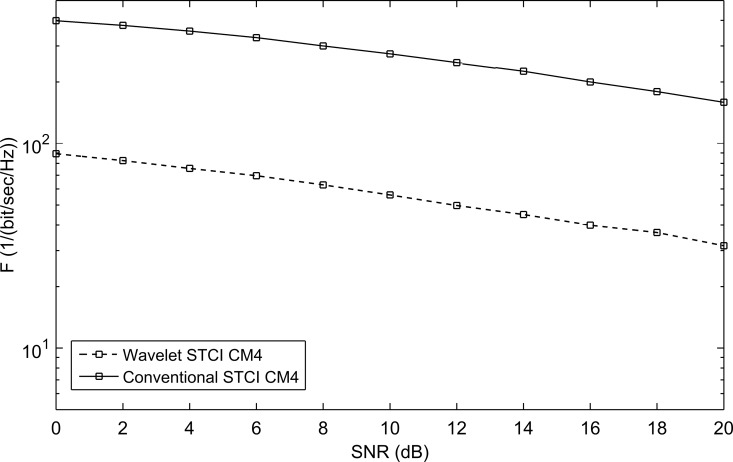
The performance of conventional STC and WSTC-I UWB system using Coiflet 3 MW and *L* = 4 Rake receiver for the CM4 model.

The performance is also examined with different non-line-of-sight (NLOS) channels CM2, CM3, and CM4 presented in the IEEE802.15.3a proposal. The WSTC-I also succeeded to enhance the performance for the non-line of sight (NLOS) channel models CM2, CM3, and CM4. This is estimated from Fig 11, Fig 12 and Fig 13 that illustrates the performance of WSTC-I for the different channel models CM2, CM3 and CM4 respectively for *L* = 4 Rake fingers. The enhancement in performance is about *ΔF* = 320/(bit/sec/Hz) at SNR = 10dB for CM2, CM3 and CM4.

### WSTC-II Results

**Fig 14:**
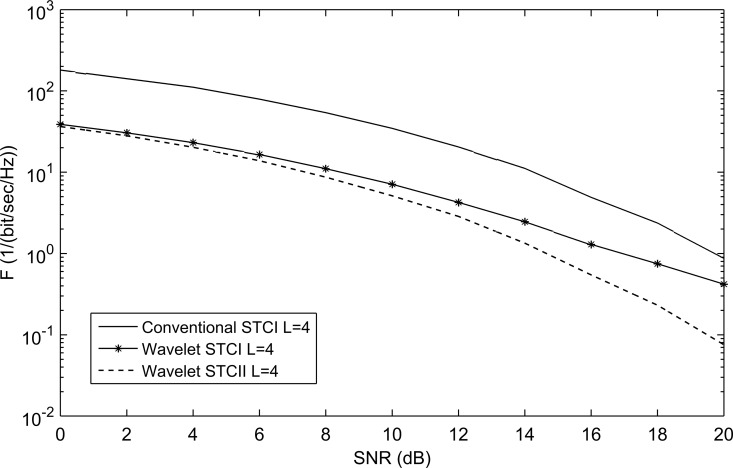
The performance of conventional STC, WSTC-I and WSTC-II UWB system using Coiflet 3 MW and L = 4 Rake receiver.

**Fig 15:**
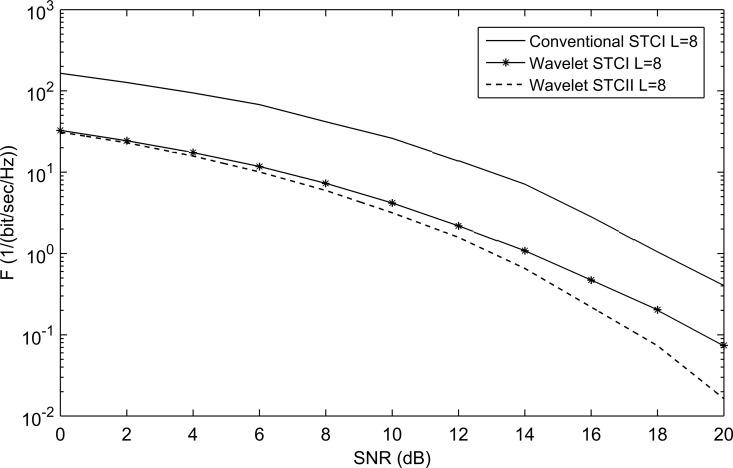
The performance of conventional STC, WSTC-I and WSTC-II UWB system using Coiflet 3 MW and L = 8 Rake receiver.

The WSTC-I scheme makes use of the frequency and the spatial multiplexing, but does not use the spatial diversity. The WSTC-II uses the benefits of the WSTC-I in addition to, the use of spatial diversity which enhances the performance and decreases the WSTC-I receiver complexity, although more complex combining process is required than the WSTC-I. Fig 14 demonstrates the performance of the WSTC-II compared with the WSTC-I and conventional STC for *L* = 4. Fig 14 shows that for *L* = 4, the WSTC-II outperforms WSTC-I by about 1.7dB and the conventional STC by 5.8dB. Fig 15 shows the performance for *L* = 8, where the performance is enhanced for the three systems (due to the increase in multipath diversity) over those with *L* = 4. It is also shown in Fig 15 that for *L* = 8, the WSTC-II outperforms WSTC-I by approximately 1.1dB and the conventional STC by 5dB.

**Fig 16:**
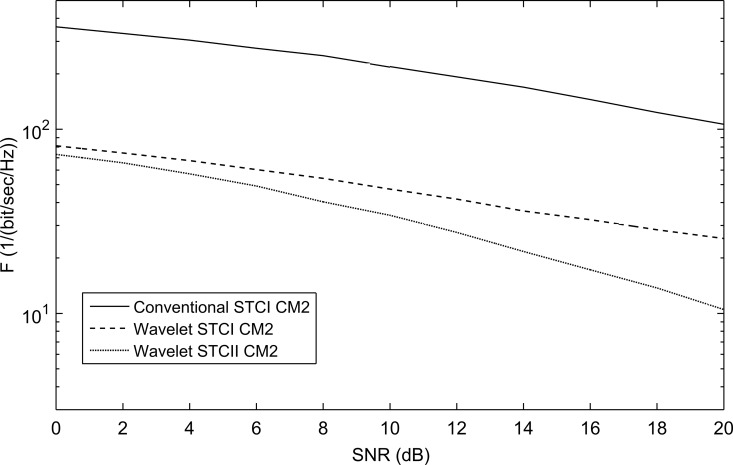
The performance of conventional STC, WSTC-I and WSTC-II UWB system using Coiflet 3 MW and *L* = 4 Rake receiver for the CM2 channel model.

**Fig 17:**
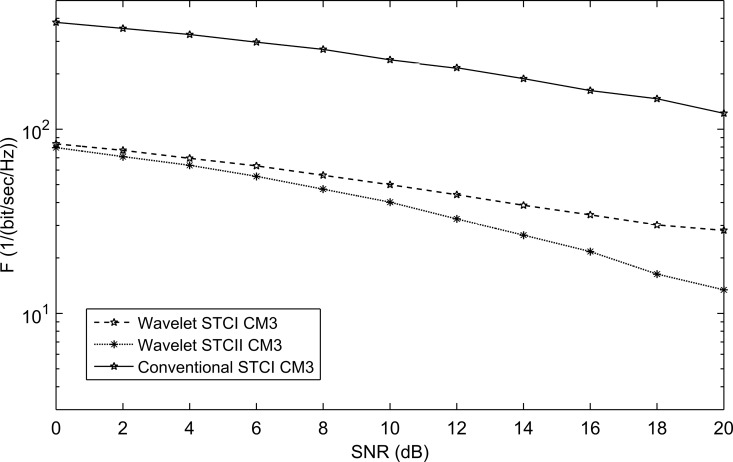
The performance of conventional STC, WSTC-I and WSTC-II UWB system using Coiflet 3 MW and *L* = 4 Rake receiver for the CM3 channel model.

**Fig 18:**
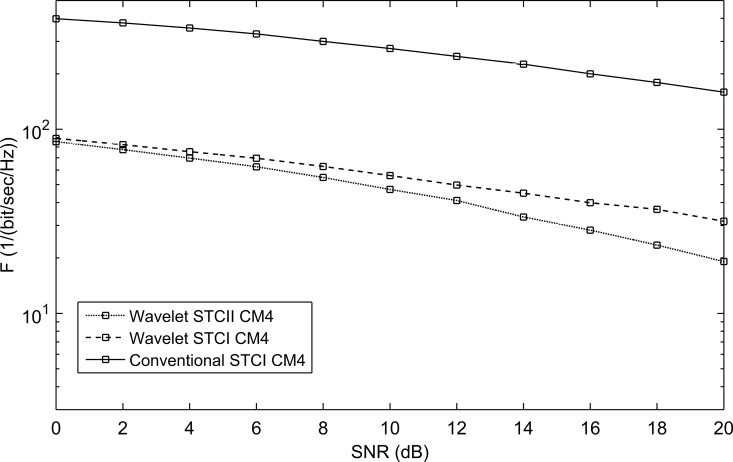
The performance of conventional STC, WSTC-I and WSTC-II UWB system using Coiflet 3 MW and *L* = 4 Rake receiver for the CM4 channel model.

For the different channel models, WSTC-II also performs better than WSTC-I and the conventional STC as illustrated in Fig 16, Fig 17 and Fig 18. Fig 16, Fig 17 and Fig 18 presents the *F* performance over the channel model CM2, CM3, and CM4 respectively, for the three MISO systems, the conventional STC, the WSTC-I and WSTC-II. It is concluded from the three figures that WSTC-II outperforms the WSTC-I by about ΔF = 10/(bit/sec/Hz) and outperforms the conventional STC by about ΔF = 330/(bit/sec/Hz) at 10 dB SNR for CM2, CM3, and CM4.

## Conclusion

In this paper two wavelet space-time coding techniques WSTC-I and WSTC-II are introduced. The two techniques multiplex different symbols in the wavelet domain of the UWB pulses and transmit the multiplexed symbols on multiple transmitting antennas to offer spatial multiplexing. The proposed schemes introduce an increase in the transmission rate to four times that of the conventional STC as a result of the multiplexing in the wavelet domain. The performances of the proposed techniques were measured using the figure of merit *F* due to the difference in data rate of the proposed and conventional techniques. The simulation results showed that WSTC schemes lead the conventional STC in performance over different channel models CM1, CM2, CM3, and CM4. The simulations also showed that the WSTC-I for CM1 outperforms the conventional STC, even with less number of fingers, thus, the best performance is obtained by a reduction in the receiver complexity. The enhancement in performance takes place due to the high correlation of the MW used with the transmitted signal and its low correlation with the noise signal especially after smoothing the received signal with WT at the receiver.

The second proposed scheme WSTC-II makes use of the same benefits of the WSTC-I in addition to introducing a spatial diversity gain and simpler combination process at the receiver. The WSTC-II simulations presented showed that the WSTC-II outperforms the WSTC-I and the conventional STC for different channel models. For CM1 and *L* = 4, the WSTC-II outperforms WSTC-I by about 1.7dB and the conventional STC by 5.8dB The WSTC-II also decreases the complexity of the receiver used in WSTC-I to the half due to its simple combination process at the receiver.
